# Phenolic Profile and Antioxidant Potential of Leaves from Selected *Cotoneaster* Medik. Species

**DOI:** 10.3390/molecules21060688

**Published:** 2016-05-26

**Authors:** Agnieszka Kicel, Piotr Michel, Aleksandra Owczarek, Anna Marchelak, Dorota Żyżelewicz, Grażyna Budryn, Joanna Oracz, Monika Anna Olszewska

**Affiliations:** 1 Department of Pharmacognosy, Faculty of Pharmacy, Medical University of Lodz, 1 Muszynskiego, 90-151 Lodz, Poland; piotr.michel@umed.lodz.pl (P.M.); aleksandra.owczarek@umed.lodz.pl (A.O.); anna.marchelak@umed.lodz.pl (A.M.); monika.olszewska@umed.lodz.pl (M.A.O.); 2 Institute of Food Technology and Analysis, Faculty of Biotechnology and Food Science, Lodz University of Technology, 4/10 Stefanowskiego, 90-924 Lodz, Poland; dorota.zyzelewicz@p.lodz.pl (D.Ż.); grazyna.budryn@p.lodz.pl (G.B.); joanna.oracz@p.lodz.pl (J.O.)

**Keywords:** *Cotoneaster*, antioxidant activity, UHPLC-PDA-ESI-QTOF-MS, HPLC-PDA, phenolic profile, phenolic, proanthocyanidin, chlorogenic acid isomer and flavonoid contents

## Abstract

The antioxidant efficiency of 70% aqueous methanolic extracts from the leaves of twelve selected *Cotoneaster* Medik. species was evaluated using four complementary *in vitro* tests based on SET- (single electron transfer) and HAT-type (hydrogen atom transfer) mechanisms (DPPH, FRAP, O_2_^•−^ and H_2_O_2_ scavenging assays). The samples exhibited the dose-dependent responses in all assays with activity parameters of EC_50_ = 18.5–34.5 µg/mL for DPPH; 0.9–3.8 mmol Fe^2+^/g for FRAP; SC_50_ = 27.7–74.8 µg/mL for O_2_^•−^; and SC_50_ = 29.0–91.3 µg/mL for H_2_O_2_. Significant linear correlations (|*r*| = 0.76–0.97, *p* < 0.01) between activity parameters and total contents of phenolics (5.2%–15.4% GAE) and proanthocyanidins (2.1%–15.0% CYE), with weak or no effects for chlorogenic acid isomers (0.69%–2.93%) and total flavonoids (0.28%–1.40%) suggested that among the listed polyphenols, proanthocyanidins are the most important determinants of the tested activity. UHPLC-PDA-ESI-QTOF-MS analyses led to detection of 34 polyphenols, of which 10 B-type procyanidins, 5 caffeoylquinic acids and 14 flavonoids were identified. After cluster analysis of the data matrix, the leaves of *Cotoneaster zabelii*, *C. splendens*, *C. bullatus*, *C. divaricatus*, *C. hjelmqvistii* and *C. lucidus* were selected as the most promising sources of natural antioxidants, exhibiting the highest phenolic levels and antioxidant capacities, and therefore the greatest potential for pharmaceutical applications.

## 1. Introduction

The oxidative stress arising from an imbalance in the total antioxidant status of the human body is believed to be involved in pathogenesis of chronic diseases usually referred to as civilization diseases, including cardiovascular and neurodegenerative disorders, cancer, metabolic syndrome, diabetes and digestive diseases [1]. Besides of endogenous defense systems, dietary antioxidants, mainly plant polyphenols, play an important role in protecting the living cells against prooxidant factors, both metabolic and external (environmental) [2]. Typical diet of Western societies, which are bearing an increasingly heavy burden of civilization diseases, is, however, poor in polyphenols and in turn rich in prooxidant xenobiotics such as hydrogenated (trans) fats, synthetic colorants and preservatives and iron/cooper-contaminated products coming from highly processed foods. The polyphenolic supplementation is therefore a generally accepted idea, which led in recent years to intensive search for plant-based, potent antioxidants active in prevention of chronic diseases. On the other hand, although a variety of plants have been studied for antioxidant activity and phenolic content, only few have been found to be sufficiently rich in low-molecular weight polyphenols characterized by high antioxidant potential and good bioavailability in internal applications [3,4]. Among plant families taxonomically featured by high content of low-molecular weight phenols, the Rosaceae family appears to be one of the most promising for future research. Apart from *Aronia*, *Crataegus*, *Malus* or *Rubus* species [5,6,7,8], widely known as functional or medicinal plants, the family comprises representatives being to date in traditional use only, but also worth of investigation.

*Cotoneaster* Medik. is a rosaceous genus of *ca*. 500 species of shrubs or small trees, occurring primarily in temperate regions of Eurasia with their center of diversity in southwestern China and the Himalayas [9]. In Poland this genus is represented by four species (*C. integerrimus* Medik*.*, *C. melanocarpus* Lodd. ex C.K. Schneid., *C. tomentosus* Lindl., *C. lucidus* Schltdl.), whose natural range covers mainly the south-eastern region of the country. Many *Cotoneaster* taxons, both native and alien, are worldwide cultivated as ornamental plants, especially appreciated for their attractive leaves turning reddish-purple in autumn and decorative, orange, red or nearly black fruits [10,11]. Several species, e.g., *C. melanocarpus* Lodd., *C. nummularia* Fisch. et Mey, and *C. tricolor* Pajork, are used in traditional medicine, especially in Iran, Turkey, Mongolia and Tibet, to treat nasal hemorrhage, excessive menstruation, hematemesis, neonatal jaundice, fever and cough [12,13,14]. In different *in vitro* tests, they are reported to have antibacterial, anti-plasmodial, anti-cholinesterase, anti-tyrosinase, antioxidant, anti-dyslipidemic, anti-glucosidase, anti-amylase, anti-diabetic, hepatoprotective and cytotoxic activities [13,15,16,17,18]. Most of these bioactivities can be attributed to the presence of low-molecular weight polyphenols including simple phenolic acids, flavonoids [19,20,21,22,23], dibenzofuran derivatives [24,25], and dimeric proanthocyanidins [26]. Metabolites of this type were isolated from twigs, leaves or roots of several species known for their ethnopharmacological significance such as *C. orbicularis* Schlecht. [19], *C. simonsii* Baker [20], *C. thymaefolia* Hort [21], *C. racemiflora* Desf [22,27], *C. acuminatus* vern. Ruins [23] and *C. horizontalis* Decne [28]. Despite the fact that polyphenols are dominant constituents of the *Cotoneaster* species, the information on the relationship between their presence and antioxidant activity is scarce and limited to a few species [12,16,18]. As reported by Zangin *et al.* [16], the total phenolics of *C. nummularia* twigs of Turkish origin varied, depending on extract polarity, from 81 to 266 mg GAE/g of the extract and were positively correlated with their antioxidant abilities, assayed by several *in vitro* tests. It follows that a considerably high phenolic content can be expected for plant tissues including leaves derived from other *Cotoneaster* species. For this reason, the objectives of this study were to screen the leaves of twelve selected *Cotoneaster* species with respect to their phenolic profile and antioxidant activity. The plant extracts were assayed using four complementary *in vitro* test systems of both single electron transfer (SET) and hydrogen atom transfer (HAT) reaction mechanisms. To identify the compounds responsible for the tested activity, the qualitative and quantitative phenolic profiles of the samples were monitored by UHPLC-PDA-ESI-QTOF-MS, HPLC-PDA and by UV-photometric methods, and the relationship between the antioxidant capacity and the phenolic content was investigated. Finally, all data were subjected to hierarchical cluster analysis to distinguish the group of species and plant materials with the greatest potential as valuable sources of natural antioxidants and candidates for *in vivo* studies of antioxidant protection.

## 2. Results and Discussion

### 2.1. Qualitative UHPLC-PDA-ESI-QTOF-MS Profiling of Cotoneaster Leaf Phenolics

**Figure 1:**
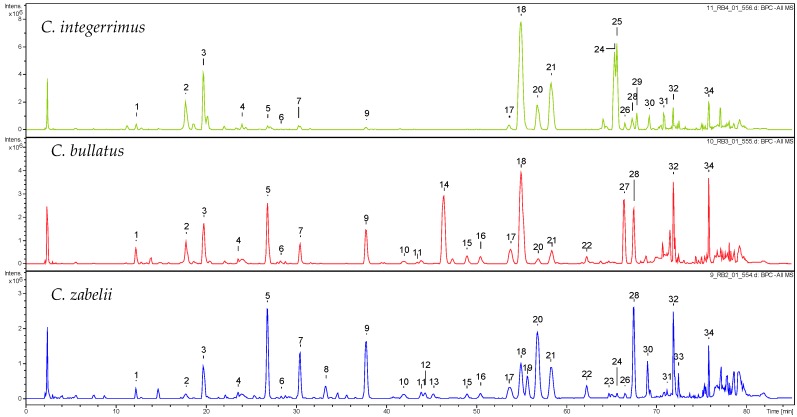
Representative total ion chromatograms (TIC) of the *C. integerrimus*, *C. bullatus* and *C. zabelii* leaf extracts recorded in the negative ion mode. The peak numbers of compounds refer to those used in Table 1.

The qualitative UHPLC-PDA-ESI-QTOF-MS profile of the 70% aqueous methanolic extracts of the *Cotoneaster* leaves revealed the presence of over thirty phenolic compounds (UHPLC peaks 1–34), thirty-two of which were fully or tentatively identified by comparing their retention times, UV-Vis spectral data and MS profiles with those of the reference compounds and the literature data. Based on the spectral profiles (Figure 1 and Table 1; for detailed MS data see Table S1), the identified phenolics were structurally classified into three main groups comprising hydroxycinnamic acid derivatives (**1**–**4**, **6**, **8**, **29**, and **31**), proanthocyanidins (**5**, **7**, **9**–**11**, **13**, **15**–**16**, and **22**–**23**) and flavonoid glycosides (**12**, **14**, **17**–**21**, **24**–**28**, **30**, and **33**).

Compounds **1**–**4**, **6**, **8**, **29** and **31** displayed absorption maxima at 325–328 nm and UV-Vis spectra characteristic of caffeic acid derivatives. Compounds **1**, **3** and **4** based on their parent [M − H]**^−^** ions at *m*/*z* 353 and the further MS fragmentation were classified as monocaffeoylquinic acid isomers. Compounds **1** and **3** exhibited the base ions at *m*/*z* 191 and the secondary ions at *m*/*z* 179 with intensities of 55**%** and 4%, respectively, whereas compound **4** displayed distinctive base peak at *m*/*z* 173. Consequently, based on the elution order, hierarchical discrimination keys [29] and comparison with reference compounds, both commercial and prepared in our laboratory, compounds **1**, **3** and **4** were identified as 3-*O*-caffeoylquinic acid (NCHA, neochlorogenic acid), 5-*O*-caffeoylquinic acid (CHA, chlorogenic acid) and 4-*O*-caffeoylquinic acid (CCHA, cryptochlorogenic acid), among which only CHA was previously found in *Cotoneaster* species (*C. simonsii*, *C. horizontalis*, *C. melanocarpus* and *C. nummularia*) [12,16,18,21]. Compounds **2**, **29** and **31** exhibited parent ions at *m*/*z* 515, typical of dicaffeoylquinic acid isomers. Although some other fragments at *m*/*z* 353 and 255 were also observed for these compounds, their MS fragmentation pattern was not possible to assign to any particular structure [29]. Compound **8**, revealing the [M + Na − 2H]**^−^** ion at *m*/*z* 613, the deprotonated molecular [M − H]**^−^** ion at *m*/*z* 591 and a peak at *m*/*z* 179, typical of caffeic acid moiety, could be also assigned as a caffeic acid derivative. Compound **6** showed absorption maximum at 310 nm, characteristic for *p*-coumaric acid derivative. Its MS fragmentation with deprotonated molecular ion at *m*/*z* 337 and secondary peak at *m*/*z* 191 led to the identification of 5-*O*-*p*-coumaroylquinic acid [30]. In this group, compounds **1**–**4** and **6** were identified in all analyzed *Cotoneaster* species, with predominant chlorogenic acid (CHA, 3).

Compounds **5**, **7**, **9**–**11**, **13**, **15**–**16** and **22**–**23** with UV maxima at 280 nm were characterized as mono- and oligomeric proanthocyanidins. Compound **7** exhibited parent [M − H]**^−^** ion at *m*/*z* 289 and by comparison with the reference compound it was identified as (−)-epicatechin, previously reported in *C. nummularia*, *C. orbicularis* and *C. accuminaus* [16,19,23]. Compounds **5** and **22** revealed pseudomolecular ions at *m*/*z* 577 and the secondary peaks at *m*/*z* 451, 425, 407 and 289 characteristic for procyanidin B-type dimers [31]. As confirmed by comparison with the authentic standard, compound **5** was identified as procyanidin B-2. In the case of compounds **9**, **11**, and **23**, their pseudomolecular ions at *m*/*z* 865 yielding the fragments at *m*/*z* 713, 577, 451, 407 and 289 indicated the presence of procyanidin B-type trimers [31]. By direct comparison with the reference standard, compound **9** was confirmed as procyanidin trimer C-1. Compounds **10** and **13**, based on MS fragmentation of parent ions at *m*/*z* 1153 to characteristic peaks at *m*/*z* 1027, 863, 575, 407 and 289, were assigned as procyanidin B-type tetramers. Compounds **15** and **16** were tentatively proposed to be (−)-epicatechin derivatives, because their [M − H]**^−^** ions at *m*/*z* 739 yielded the fragments at *m*/*z* 289, characteristic for epicatechin, the dominant unit in the B-type oligomeric procyanidins of the analyzed *Cotoneaster* species. In consequence, in the group of proanthocyanidins, 10 individual compounds were identified with the predominant dimer B-2 and trimer C-1 detected in all analyzed *Cotoneaster* samples.

Compounds **12**, **14**, **17**–**21**, **24**–**28**, **30** and **33**, based on the UV-Vis spectra with two maxima, first at 260–280 and second at 345–355 nm, were classified as flavonoids. All compounds in this group were found to be flavonoid mono- and diglycosides due to neutral losses in their MS spectra of sugar moieties (−132 for pentose, −162 for hexose and −146 for rhamnose). Furthermore, the signals in the MS spectra, assignable to the aglycone moieties at *m*/*z* 300 or 285 suggested that all detected flavonoids are glycosides of quercetin or kaempferol. 

Compounds **18** and **21** with the pseudomolecular [M − H]**^−^** ions at *m*/*z* 463 were identified with the authentic standards as hyperoside and isoquercitrin, respectively. These flavonoids were previously reported as constituents of *C. melanocarpus* and *C. orbicularis* [12,19]. Compound **28** revealed [M − H]^−^ ion at *m*/*z* 447 and after comparison with the standard was assigned as quercitrin, previously reported in *C. thymaefolia* [21]. Compounds **24**–**25** and **30**, based on their pseudomolecular ions at *m*/*z* 505 which yielded fragments at *m*/*z* 463 and 300, were tentatively assigned as quercetin hexoside derivatives. Compound **14** as a flavonoid diglycoside, with the parent ion at *m*/*z* 595 revealing neutral loss of 132 and 162 amu characteristic for pentose and hexose, respectively, was identified with the authentic standard as quercetin 3-*O*-(2′′-*O*-β-xylosyl)galactoside. Compounds **12**, **17**, **20** and **27** exhibited pseudomolecular ions at *m*/*z* 609. By direct comparison with the reference standards, compounds **12** and **20** were identified as quercetin 3-*O*-β-glucoside-7-*O*-α-rhamnoside and rutin, respectively. Among the latter compounds, only rutin was previously detected in *C. melanocarpus*, *C. orbicularis* and *C. thymaefolia* [12,19,21]. Compounds **17** and **27** were tentatively assigned to quercetin rhamnoside-hexoside due to the neutral losses of rhamnose (−146 amu) and hexose (−162 amu) and production of aglycone peaks at *m*/*z* 300. Compounds **19**, **26** and **33** with parent ions at *m*/*z* 593 were tentatively identified as quercetin or kaempferol diglycosides. Compound **19** was assigned as quercetin dirhamnoside due to the observed fragments ion at *m*/*z* 447 and the aglycone signal at *m*/*z* 300. In the case of compounds **26** and **33**, fragmentation of the parent ions led to the cleavage of rhamnose (−146 amu) and hexose (−162 amu) resulting in production of the aglycone peaks at *m*/*z* 285. Thus, compounds **26** and **33** were proposed as kaempferol rhamnoside-hexosides. In all analyzed *Cotoneaster* species, flavonoids were mainly represented by hyperoside (**18**), isoquercitrin (**21**), rutin (**20**) and quercetin rhamnoside-hexoside (**17**).

### 2.2. Quantitative Determination of Phenolics, Proanthocyanidins, Flavonoids and Chlorogenic Acid Isomers in the Cotoneaster Leaf Extracts

Total phenolic content (TPC) of the *Cotoneaster* leaf samples was determined by the FC assay and expressed as Gallic acid equivalents (GAE). In this method, the reagent is formed from a mixture of phosphotungstic and phosphomolybdic acids, which after oxidation of the phenols were reduced to blue oxides of tungsten and molybdenum. Although the FC assay is widely used to estimate total phenolics in a sample matrix, it can be also useful as a nonspecific antioxidant assay, because its basic redox mechanism is similar to those occurring in antioxidant tests [32,33].

**Figure 2:**
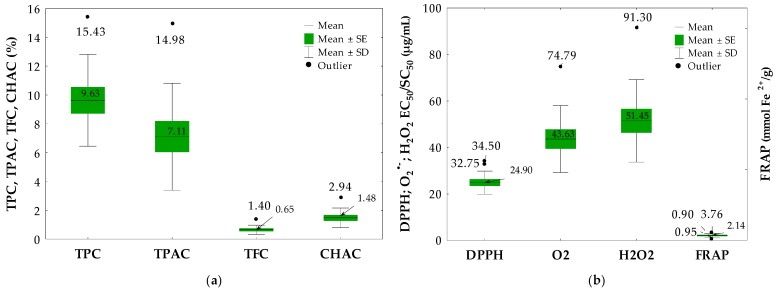
Box-whisker plot analysis of: (**a**) total contents of phenolics (TPC), proanthocyanidins (TPAC) flavonoids (TFC) and chlorogenic acid isomers (CHAC); and (**b**) antioxidant and reducing activity tested by SET and HAT-type methods in the *Cotoneaster* leaf samples. Mean values ± standard errors (SE) and standard deviation (SD).

As shown in Table 2 and Figure 2a, the total phenolic content (TPC) in the *Cotoneaster* leaves varied from 5.2% to 15.4% GAE of the leaf dry weight (dw), depending on the tested species, with the average value of 9.6% GAE. The highest TPC levels were observed for the leaf sample of *C. bullatus*, whereas *C. melanocarpus* and *C. tomentosus* contained the lowest amounts of phenolics. With the levels about or above 10% GAE, six samples may be classified as polyphenol-rich and especially promising for further studies of antioxidant activity. Within this group, the TPC values decreased in the order: *C. bullatus* > *C. zabelii* > *C. hjelmqvistii* > *C. divaricatus* > *C. lucidus* > *C. splendens*.

According to the literature, the quantitative data regarding *Cotoneaster* polyphenols are limited only to a few studies on the dry extracts and indicate a large discrepancy in polyphenolic contents between the species [16,18]. According to Zengin *et al.* [16], different polarity extracts from the twigs of *C. nummularia* collected in Turkey contained total phenolics in the range 8.1%–26.6% GAE, with the highest level found for methanol and water extracts. On the other hand, as reported by Mohamed *et al.* [18], the TPC value of methanol extract from *C. horizontalis* branches cultivated in Egypt amounted only 1.4% GAE. It is thus clear, that the *Cotoneaster* leaves of Polish origin are particularly rich in polyphenols, whose contents of up to 15% GAE in dried plant materials are comparable or even higher than those observed for other polyphenol abundant Rosaceae leaves, *i.e.*, *Rosa canina* (10.0%–15.2% GAE) [34], various Sorbus species (5.1%–12.3% GAE) [35], *Aronia melanocarpa* (13.9% GAE) [36] and *Ribes nigrum* (3.2%–4.4% GAE) [37]. 

**Figure 3:**
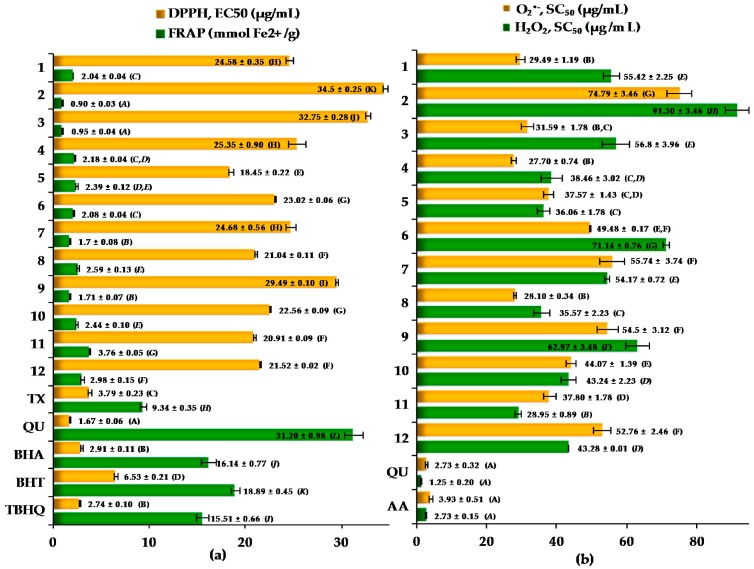
Antioxidant activity of the *Cotoneaster* leaf extracts and standard antioxidants in: (**a**) the DPPH and FRAP tests; and (**b**) O_2_^•−^ and H_2_O_2_ scavenging tests. Results are presented as the mean values ± SD (*n* = 3); for each activity parameter different capital letters (A–G and A–L) indicate significant differences at *p* < 0.05 by Tukey’s test; scavenging ability (EC_50_ and SC_50_), the amount of the plant samples or the standards required for 50% reduction of initial ROS concentration; FRAP, ferric reducing antioxidant power expressed in mmol Fe^2+^ per g of the dry leaves or the reference compounds; for the species codification see Table 1; reference standards: TX, Trolox^®^; QU, quercetin; AA, ascorbic acid; BHA, butylated hydroxyanisole; BHT, 2,6-di-*tert*-butyl-4-methylphenol; TBHQ, *tert*-butyl-hydroquinone.

It is obvious, that the total phenolic level as measured by FC method does not give a full picture of the quality and quantity of the phenolic compounds in the matrix. There may be some interferences resulting in over- or underestimation and arising from non-phenolic reducing constituents, e.g., sugars, aromatic amines or ascorbic acid, as well as from differences in molecular structures and reactivity between original plant polyphenols and gallic acid as a calibrating standard [34]. Thus, to better characterize the phenolic profile of the *Cotoneaster* leaves, complementary assays of their total proanthocyanidins (TPAC), chlorogenic acid isomers (CHAC) and flavonoids (TFC) were conducted. The TPAC was determined by spectrophotometric *n*-butanol/HCl method and expressed in cyanidin chloride equivalents (CYE). The CHAC as a sum of monocaffeoylquinic acid isomers (NCHA, CHA and CCHA) and TFC after acid hydrolysis as a sum of flavonoid aglycones were quantified using RP-HPLC-PDA methods. As shown in Table 2 and Table 3, and Figure 3a, majority of the tested samples exhibited relatively high levels of total proanthocyanidins ranging from 2.6% to 15.0% CYE dw, with the average value of 7.1% CYE. The highest TPAC contents were noted for *C. bullatus* leaves (15.0% CYE), followed by *C. zabelii* (10.9% CYE), *C. hjelmqvistii* (9.4% CYE), *C. splendens* and *C. divaricatus* (for each of about 9.1% CYE). It is noteworthy, that the content of proanthocyanidins in all samples tested had a strong impact on their total phenolic level, which is reflected by high and statistically significant correlation found between these values (|*r*| (*R*^2^) = 0.9419 (0.8872), *p* < 0.001). With the TPAC levels constituting 39%–97% of the TPC values, proanthocyanidins appear to be the dominant phenolic constituents of the tested leaves. The content of caffeoylquinic acid isomers varied among the species (0.69%–2.93% dw), with the highest amount found for *C. hjelmqvistii* and *C. divaricatus* (2.9% and 2.1%, respectively). The chlorogenic acid (CHA) was the dominant isomer in all tested samples, and its level constituted 73.3%–92.1% of the sum of three quantified isomers. The highest contents of two other isomers were found in the *C. divaricatus* sample, with the levels of 0.33% for NCHA and 0.10% for CCHA. Flavonoids as the third analyzed group also revealed high quantitative variability with the TFC levels ranging from 0.3% to 1.4% dw, depending on the sample. The leaves of *C. integerrimus* exhibited the highest TFC content and those of *C. melanocarpus* the lowest. As regards individual aglycones, quercetin and kaempferol were detected in majority of the *Cotoneaster* leaf hydrolyzates (Table 2). All analyzed samples were devoid of other flavonoid aglycones *i.e.*, apigenin and luteolin reported earlier [16,19,21,23] for the twigs, leaves and roots of *C. nummularia*, *C. orbicularis*, *C. thymaefolia* and *C. acuminatus*. Out of flavonoids detected in the assayed samples, the most abundant aglycone was quercetin with the levels (0.26%–1.32% dw) constituting 68%–100% of the sum of both quantified aglycones. In ten samples, quercetin was accompanied by kaempferol, whose content reached up to 0.26% dw, whereas, in the samples of *C. nanshan* and *C. bullatus*, the quercetin at the level of 0.97% and 0.61%, respectively, was the single flavonoid found.

### 2.3. Antioxidant Activity of the *Cotoneaster* Leaf Extracts

A variety of antioxidant assays based on hydrogen atom transfer (HAT) and/or single electron transfer (SET) reaction mechanisms have been widely employed for quantification of antioxidant capacity of phenolic samples, but there is no universal assay that can accurately reflect all the antioxidants in a complex system [32]. Therefore, to evaluate the total antioxidant capacity of plant matrix it is necessary to diversify the tests. In this study, four in vitro assays, namely the two widely used SET-based methods: the 2,2-diphenyl-1-picrylhydrazyl (DPPH) free radical scavenging and the ferric reducing antioxidant power (FRAP) tests as well as two HAT-based methods for scavenging superoxide anion (O_2_^•−^) and hydrogen peroxide (H_2_O_2_) were employed to evaluate the antioxidant properties of the *Cotoneaster* leaf extracts. In all applied tests, on the basis of the chemical reaction involved, the extract capability was evaluated for free radical scavenging (DPPH, O_2_^•−^), for quenching non-radical ROS (H_2_O_2_) as well as for reducing metal ions (Fe^3+^).

As presented in Figure 2b and Figure 3a,b, activity of the analyzed *Cotoneaster* leaf extracts increased in a concentration-dependent manner with different range of efficiencies depending on the test. In the DPPH assay, the EC_50_ values of all tested samples varied in a narrow range of 18.5–34.5 µg/mL. The highest activity was noted for leaf extract of *C. divaricatus* followed by that of *C. bullatus*, *C. hjelmqvistii* and *C. zabelii*. In the FRAP test, the range of FRAP values for the tested samples was relatively wider (0.9–3.8 mmol Fe^2+^/g), and the highest activity was found for *C. bullatus*, *C. zabelii* and *C. hjelmqvistii* samples. In the H_2_O_2_ test, the differences in the SC_50_ values between the samples were also pronounced (29.0–91.3 µg/mL) and the highest activity parameters were obtained for the samples of *C. bullatus*, *C. hjelmqvistii* and *C. divaricatus*. It is of note that the leaves of *C. bullatus*, *C. zabelii*, *C. divaricatus* and *C. hjelmqvistii*, found in the present study as the richest sources of polyphenols, also displayed the highest antioxidant and reducing activities in aforementioned tests.

In the case of the O_2_^•−^ test, the SC_50_ values also varied in wide range of 27.7–74.8 µg/mL but a different order of the antiradical efficiencies was observed among the samples. The leaves of *C. lucidus*, *C. hjelmqvistii* and *C. integerrimus* displayed higher activity (mean values of SC_50_ = 28.4 µg/mL) in comparison to the leaf samples of *C. divaricatus* (SC_50_ = 37.6 µg/mL), *C. bullatus* (SC_50_ = 37.8 µg/mL) and *C. zabelii* (SC_50_ = 52.8 µg/mL), for which the highest capacity was observed in the DPPH, FRAP and H_2_O_2_ tests. The relatively high O_2_^•−^ scavenging activity of the *C. integerrimus* leaves, connected with only moderate TPC level as compared to *C. lucidus* and *C. hjelmqvistii* samples, may be influenced by the considerably higher flavonoid content (1.4% dw). It is known, that flavonoids as hydrogen atom donators are able to effectively reduce highly oxidizing radicals such as superoxide, peroxyl, alkoxyl, and hydroxyl radicals [38], which was also proven in the present study for the reference quercetin (SC_50_ = 2.7 µg/mL). 

The literature data on the antioxidant activity of *Cotoneaster* species are scarce and present highly variable results [12,16,17]. In the DPPH scavenging test, the dry ethanol extract from *C. horizontalis* branches was characterized by the EC_50_ value of 19.5 μg/mL [17]. On the other hand, the dry methanol extracts of *C. nummularia* twigs and *C. melanocarpus* leaves exhibited notably lower activity, with the EC_50_ values of 104 and 106 μg/mL, respectively [12,16]. As each study focuses only on one species at a time, it is difficult to draw any valid conclusions about the reasons of this variability. Our research is the first to compare larger number of *Cotoneaster* species in terms of their antioxidant capacity in one study design and therefore provides better insight into the nature and extent of the activity of the genus.

As presented in the Table 4, the antioxidant activity of all *Cotoneaster* leaf samples was strongly dependent on the total polyphenol (TPC) and proanthocyanidin (TPAC) contents, which is reflected in significant linear correlations (*p* < 0.01) between the TPC, TPAC values and the results of DPPH, FRAP and H_2_O_2_ reduction tests. On the other hand, no such effect was observed in case of TFC and CHAC which was caused probably by the relatively lower level of flavonoids and chlorogenic acid isomers in comparison to proanthocyanidins. The significant correlations were also found between the results of three aforementioned antioxidant activity assays, that suggests that the *Cotoneaster* extracts could be considered universal antioxidants utilizing both basic reaction mechanisms and acting as direct ROS quenchers as well as reducing agents. Contrarily, the results of O_2_^•−^ scavenging assay did not correlate neither with quantitative findings nor with the activity of the samples in the SET-type tests performed. Significant correlation was found only with the results of H_2_O_2_ quenching test, utilizing HAT-type mechanism. This phenomenon could be affected by non-phenolic constituents, which could co-occur in the *Cotoneaster* extracts. However, a more detailed study addressing the chemical nature of these compounds and their presence in the *Cotoneaster* samples is required. 

Aside from the O_2_^•−^ scavenging, significant, linear relationships existing between antioxidant capacity parameters and phenolic contents, suggests that polyphenols are important determinants of the SET- and HAT-type antioxidant activity of the tested *Cotoneaster* extracts. Moreover, given the more detailed quantitative analysis and the respective correlation results, proanthocyanidins seem to be the major contributors to the observed capacities. As was proven in numerous *in vitro* systems [39], the antioxidant activity of this group of compounds is largely dependent on their molecular structure, particularly the molecular weight expressed as degree of polymerization (DP). It was demonstrated, that proanthocyanidin oligomers characterized by DP < 10 are especially effective ROS/RNS scavengers, with the antioxidant power greater than that of vitamins C and E [40]. Considering the high extraction efficiency of hydromethanolic solutions towards small proanthocyanidin oligomers [41], it can be safely assumed that the proanthocyanidin fraction of the investigated *Cotoneaster* extracts is dominated by this type of compounds. Proanthocyanidins including B-type dimers, trimers and tetramers were indeed identified in the extracts by UHPLC-QTOF-MS (Table 1). Among highly valued plant materials, characterized by especially high content of low molecular proanthocyanidin oligomers are seeds of *Vitis vinifera* [41]. Broadly acknowledged antioxidant activity of proanthocyanidin-rich grape seed extract, reflected in strong inhibition of oxidation of lipid and low density lipoproteins, is at the heart of its many health benefits, including cardioprotective and anti-atherosclerotic effect [41,42]. Thus, other sources of this valuable group of metabolites such as investigated *Cotoneaster* leaves definitely deserve attention and further studies.

### 2.4. Hierarchical Cluster Analysis of the Phytochemical and Activity Data

An agglomerative hierarchical cluster analysis (HCA) was performed for all experimental data, taking the Euclidean distance as metric and the complete linkage method as an amalgamation rule. 

**Figure 4:**
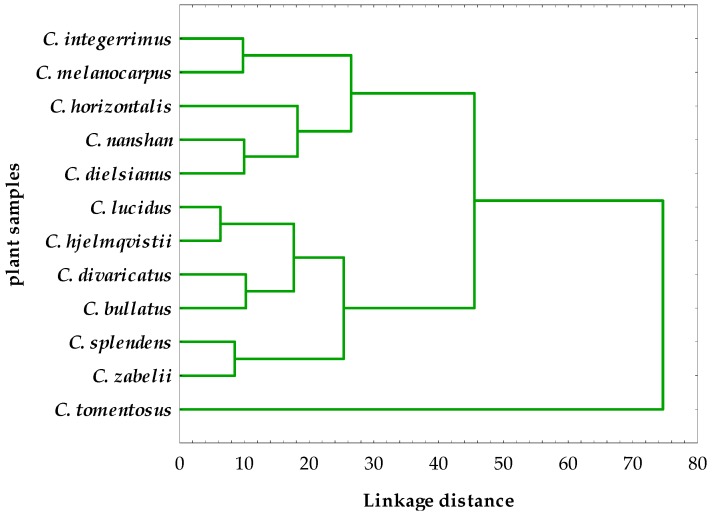
Hierarchical cluster analysis with all variables obtained for the *Cotoneaster* leaves using the complete linkage method and Euclidean squared distance.

As shown in Figure 4, the analyzed *Cotoneaster* leaves were divided into three distinct clusters (CL-1-CL-3), which correspond to three diverse levels of their phytochemical and antioxidant capacities. In the CL-1, the six leaf samples of *C. zabelii*, *C. splendens*, *C. bullatus*, *C. divaricatus*, *C. hjelmqvistii* and *C. lucidus* were associated with the highest content of polyphenols (the mean TPC of 12.2% ± 1.9% GAE) and proanthocyanidins (the average TPAC of 10.0% ± 2.9% CYE) as well as high antioxidant activity as reported in Figure 3. The cluster CL-2 with five samples of *C. dielsianus*, *C. nanshan*, *C. horizontalis*, *C. melanocarpus* and *C. integerrimus* was characterized by almost twice lower levels of polyphenols (the mean TPC of 7.4% ± 1.3% GAE) and proanthocyanidins (the average TPAC of 4.6% ± 1.5% CYE) and also the significantly weaker antioxidant potential in comparison to CL-1. The CL-3 includes only one species of *C. tomentosus*, due to its significantly worse quality parameters than observed for the other samples, both in the phenolic content and antioxidant ability. 

In order to more thoroughly assess the impact of phenolics on the antioxidant properties of the most valued samples grouped in the cluster CL-1, their average EC_50_ value (21.6 μg of the leaf dry weight/mL) for the DPPH test was recalculated to the phenolic effective concentration PEC_50_ with the use of the respective TPC levels. The obtained PEC_50_ value of 2.64 μg of the phenolics/mL was comparable with that measured (Figure 3) for the positive control of Trolox (EC_50_ = 3.79 μg/mL) and the reference synthetic antioxidants such as BHA (EC_50_ = 2.91 μg/mL) and TBHQ (EC_50_ = 2.74 μg/mL) widely used in food and cosmetic industry. Based on the above results, the six *Cotoneaster* samples (cluster CL-1) would be recognized as promising candidates for further *in vitro* and *in vivo* studies of antioxidant protection with great potential for the use as easily accessible, cost-effective and potentially non-toxic antioxidant agents. 

## 3. Materials and Methods 

### 3.1. Plant Material

Leaf samples of twelve selected *Cotoneaster* Medik. species were collected and authenticated in September/October 2012, in the Botanical Garden (51°45′N 19°24′E) in Lodz (Poland) or in the Arboretum (51°49′N 19°53′E), Forestry Experimental Station of Warsaw University of Life Sciences (SGGW) in Rogow (Poland). The list of the species employed, their collection sites and herbarium codes are given in Table 5. The voucher specimens were deposited in the Herbarium of the Department of Pharmacognosy, Medical University of Lodz (Poland). After harvest, the raw materials were air-dried under normal conditions, powdered with an electric grinder, sieved through a 0.315 mm sieve, and stored in airtight containers until use.

### 3.2. General

HPLC grade purity reagents and standards such as 2,2-diphenyl-1-picrylhydrazyl (DPPH), 2,4,6-*tris*-(2-pyridyl)-s-triazine (TPTZ), luminol, nitrobluetetrazolium, xanthine, xanthine oxidase, hydrogen peroxide; horseradish peroxidase, (±)-6-hydroxy-2,2,7,8-tetramethylchroman-2-carboxylic acid (Trolox^®^, TX), ascorbic acid (AA), quercetin dihydrate (QU), kaempferol (KA), Gallic acid monohydrate (GA), cyanidin chloride (CY), chlorogenic acid (CHA), hyperoside (quercetin 3-*O*-β-d-galactopyranoside), isoquercitrin (quercetin 3-*O*-β-d-glucopyranoside), procyanidins B-2 and C-1, (−)-epicatechin were purchased from Sigma-Aldrich (Seelze, Germany/St. Louis, MO, USA). The analytical grade antioxidant standards such as butylated hydroxyanisole (BHA), 2,6-di-*tert*-butyl-4-methylphenol (BHT) and *tert*-butylhydroquinone (TBHQ) were obtained from the same supplier. The qualitative standards of 3-*O*-caffeoylquinic acid (NCHA) and 4-*O*-caffeoylquinic acid (CCHA) were prepared by isomerization of CHA as described previously [43]. The standards of rutin, quercetin 3-*O*-(2′′-*O*-β-xylosyl)galactoside, quercetin 3-*O*-β-glucoside-7-*O*-α-rhamnoside and quercitrin (quercetin 3-*O*-β-rhamnoside) were previously isolated in our laboratory with at least 95% HPLC purity. HPLC grade solvents, methanol (MeOH) and *orto*-phosphoric acid (H_3_PO_4_) used for HPLC analyses were from Avantor Performance Materials (Gliwice, Poland). All other chemicals and solvents were of analytical grade and supplied by Avantor PM (Gliwice, Poland).

For nonenzymatic spectrophotometric tests, absorbance was measured using a Lambda 25 spectrophotometer (Perkin-Elmer, Walthman, MA, USA), in 10 mm quartz cuvettes. The samples for the FRAP assay were incubated in a constant temperature using a BD 23 incubator (Binder, Tuttlingen, Germany). Enzymatic tests were performed in 96-well plates and monitored using a microplate reader, Synergy 4 (BioTek, Vinooski, VT, USA). 

### 3.3. Extraction and Hydrolysis Procedures

The accurately weighed samples (100 or 500 mg) were first defatted by pre-extraction with chloroform (20 mL, 15 min, the chloroform extracts was discarded) and then refluxed for 30 min with 30 mL of 70% (*v*/*v*) aqueous methanol. After filtering the extract, the pellet was extracted twice for 15 min with 20 mL of the same solvent. The combined filtrates were diluted with the solvent to 100 mL. Each sample was extracted in triplicate to give crude fluid extracts (CE), which were tested for their total phenolic (TPC), proanthocyanidin (TPAC) and chlorogenic acid isomer (CHAC) contents as well as antioxidant activity. For qualitative UHPLC analyses, the prepared crude extracts were evaporated to dryness to obtain dry extracts (DE). The samples of DE (10 mg) were dissolved in 1 mL of 70% aqueous methanol, filtered through a PTFE syringe filter (25 mm, 0.2 μm, Vitrum, Czech Republic) and injected (3 µL) into the UHPLC system.

The flavonoid content was determined as the total content of flavonoid aglycones obtained after acid hydrolysis. The samples (500 mg) were first defatted by pre-extraction (15 min) with 20 mL chloroform, and then refluxed for 1 h with the mixture of 6 mL 36.5% (11.8 M, *w*/*v*) hydrochloric acid and 30 mL 90% (*v*/*v*) aqueous methanol. Thus the hydrolytic solution consisted of 1.97 M (70 g/L) hydrochloric acid and 75% aqueous methanol (*v*/*v*). After filtration, the sample was extracted twice with 20 mL of 90% aqueous methanol for 10 min. The combined hydrolyzates were diluted to 100 mL with methanol to obtain the hydrolyzed extract (HE). 

For quantification of flavonoid aglycones and chlorogenic acid isomers, HE and CE were filtered through a PTFE syringe filter and injected (20 µL) into the HPLC system. The determinations were performed after three separate extractions of each leaf sample, and each HE and CE were injected into the HPLC system in triplicate.

### 3.4. Phytochemical Profiling

#### 3.4.1. UHPLC-PDA-ESI-QTOF-MS and HPLA-PDA Analyses

UHPLC-PDA-ESI-QTOF-MS analyses of DE were performed on UHPLC-3000 RS system (Dionex, Dereieich, Germany) with a diode array detector with multiple wavelength (Thermo Fisher Scientific Inc., Waltham, MA, USA), and an ultrahighresolution hybrid quadrupole/time-of-flight mass spectrometer (UHR-Q-TOF–MS, Bruker Daltonics GmbH, Bremen, Germany) using an electrospray ionization (ESI) source operating in negative modes. Instrument control, data acquisition, and evaluation were done with the QTOFControl 3.2, HyStar 3.2, and Chromeleon 6.8.1 Chromatography Data System softwares, respectively. Separations were carried out on a Accucore C18 column (150 × 3.0 mm, 2.6 μm, Thermo Scientific). The mobile phase consisted of solvent A (water:formic acid, 100:0.1, *v*/*v*) and solvent B (acetonitrile:formic acid, 100:0.1, *v*/*v*) with the elution profile as follows: 0–25 min, 4%–10% (*v*/*v*) B; 25–65 min, 10%–20% B; 65–73 min, 85% B; 73–79 min, 85%–4%; 79–85 min, 4% B. The flow rate was 0.3 mL/min. The column temperature was 25°C. UV spectra were recorded over a range of 200–450 nm, chromatograms were acquired at 280, 320 and 360 nm. The LC eluate was introduced directly into the ESI interface without splitting. ESI parameters: the nebuliser pressure was 14.5 psi; dry gas flow 8 L/min; dry temperature 200°C; and capillary voltage 4.5 kV. Analysis was carried out using scan from −*m*/*z* 50 to 1500. Compounds were analyzed in negative ion mode. The MS fragmentation was obtained in Auto MS/MS mode for the most abundant ion at the time. The identification of individual phenolic compounds was achieved by comparison of their retention times, UV-Vis spectra and MS profile with those of the available reference standards and data reported in the literature.

HPLC-PDA analyses of CE and HE were performed on a Waters 600E Multisolvent Delivery System (Waters, Milford, MA, USA) with a PDA detector (Waters 2998) scanning in the wavelength range of 220–550 nm; and a manual injector rheodyne, 7725i model (Rheodyne, Pittsburgh, PA, USA) with 20 µL sample loop. The constant temperature of the column was maintained using a Jetstream Plus 5480 thermostat (Thermotechnic Products, Langenzersdorf, Austria).

#### 3.4.2. Determination of Total Flavonoid Content (TFC)

The total flavonoid content in HE was determined using HPLC-PDA (see Section 3.4.1.) by the slightly modified method [44]. The analytical column was a C18 Nucleodur C-18 Gravity (5 μm, 250 × 4.6 mm i.d.; MN, Düren, Germany), guarded by a C18 Hypersil ODS pre-column (5 μm, 4 × 4 mm i.d.; Agilent Technologies, Santa Clara, CA, USA). The analysis was performed using the mobile phase consisted of solvent A (water/orthophosphoric acid, 99.5:0.5 *v*/*w)*, and solvent B (methanol) with the elution profile as follows: 0–16 min: 45%–65% B, 16.01–18 min: 45% B (equilibration). The flow rate was 1.4 mL/min and the column temperature was set at 40 °C. The compounds were monitored at 370 nm, and their UV-Vis spectra were recorded for peak purity and identification tests. The aglycones QU and KA were identified by comparison of their UV-Vis spectra and retention times (t_R_ = 8.8 and 12.2 min, respectively) with those of authentic standards. The flavonoid content (TFC) was calculated from the calibration curves of the external standards within the concentration ranges of approximately 2.5–85.2 µg/mL for QU and 0.6–20.0 µg/mL for KA.

#### 3.4.3. Determination of Chlorogenic Acid Isomers (CHAC)

The total chlorogenic acid isomers in CE was determined by HPLC-PDA (see Section 3.4.1.) with some variations of method [35]. The analytical column was a C18 Ascentis^®^ Express (2.7 μm, 75 × 4.6 mm i.d.; Supelco, Bellefonte, PA, USA), guarded by a C18 Ascentis^®^ C18 Supelguard column (3 μm, 20 × 4 mm i.d.; Supelco). The mobile phase consisted of solvent A (water/orthophosphoric acid, 99.5:0.5 *v*/*w*) and solvent B (acetonitrile) with the elution profile as follows: 0–14 min, 6%–30% B (*v*/*v)*; 14–15 min, 30%–50% B; 15–17 min, 50% B; 17–18 min, 50%–6% B; 18–21 min, 6% B (equilibration). The flow rate was 1.4 mL/min and the column temperature was maintained at 25 °C. The chromatograms were recorded at 325 nm and the peaks of NCHA, CHA and CCHA were assigned based on the UV-Vis spectra and retention times (t_R_ = 3.6, 6.1 and 6.8 min, respectively) of the standard compounds. The contents of the isomers were calculated from the calibration curve of chlorogenic acid (CHA) obtained within the concentration ranges of 2.6–265.3 µg/mL.

#### 3.4.4. Determination of Total Phenolic Content (TPC)

The total phenolic content (TPC) in CE was determined according to the Folin–Ciocalteu (FC) method as described previously [44,45]. The results were expressed as g of Gallic acid equivalents per 100 g of dry weight of the plant material (% GAE).

#### 3.4.5. Determination of Total Proanthocyanidin Content (TPAC)

Determination of the total proanthocyanidin content (TPA) in CE was performed using the modified *n*-butanol/HCl method of Porter *et al.* [35,46]. The results were expressed as g of cyanidin chloride equivalents per 100 g of dry weight of the plant material (% CYE).

### 3.5. Antioxidant Activity Testing

#### 3.5.1. DPPH Free Radical Scavenging Assay

The DPPH scavenging activity of CE was determined according to the method optimized earlier [45]. The analytical samples were prepared using serial dilutions of CE in 70% aqueous methanol (*v*/*v*) to obtain the plant sample concentration of 13–40 μg/mL in the reaction medium. Finally, the EC_50_ values were calculated from five-point calibration curves by plotting the sample concentration in the reaction medium (μg/mL) *versus* the concentration (μg/mL) of remaining DPPH radical as determined from the DPPH calibration curve.

#### 3.5.2. Ferric Reducing Antioxidant Power (FRAP) Assay

The determination of ferric reducing ability (FRAP) of CE was performed based on the original method [47] with slight modifications [48]. Prior to the analysis, the CE was diluted with 70% aqueous methanol (*v*/*v*) to the plant sample concentration of 5–10 μg/mL in the reaction medium. The activity was expressed in millimoles of Fe^2+^ produced by 1 g of the dry plant material as calculated from the calibration curve of ferrous sulfate.

#### 3.5.3. Superoxide Anion Radical (O_2_^•−^) Scavenging Assay

The capacity of CE to scavenge superoxide anion radical (O_2_^•−^) was monitored using the xanthine/xanthine oxidase system with nitrobluetetrazolium (NBT) as described previously [49]. Before testing, the CE was evaporated to dryness and redissolved in PBS solution (without Ca^2+^, Mg^2+^) to obtain the plant sample concentration of 15–100 μg/mL in the reaction medium. In order to evaluate the possibility of direct interaction of CE with the enzyme, the uric acid production by xanthine oxidase was monitored at 295 nm [49] and no inhibitory effect on the enzyme was observed. The SC_50_ values for scavenging capacity were calculated from five-point calibration curves by plotting the sample concentration in the reaction medium (μg/mL) *versus* the percentage of O_2_^•−^ radical.

#### 3.5.4. Hydrogen Peroxide (H_2_O_2_) Scavenging Assay

The ability of CE to scavenge hydrogen peroxide (H_2_O_2_) was determined according to the chemiluminescence method as described previously [49]. Before testing, the CE was evaporated to dryness and redissolved in PBS solution (without Ca^2+^, Mg^2+^) to obtain the sample concentration of 15–100 μg/mL in the reaction medium. The SC_50_ values were calculated from five-point calibration curves by plotting the sample concentration in the reaction medium (μg/mL) *versus* the percentage of H_2_O_2_.

### 3.6. Statistical and Data Analysis

The results were expressed as means ± standard deviation (SD) of triplicate determinations. The statistical analyses (calculation of SD, one-way analysis of variance, HSD Tukey tests, linearity studies and hierarchical cluster analysis) were performed using the Statistica12Pl software for Windows (StatSoft Inc., Krakow, Poland), with *p* values less than 0.05 being regarded as significant.

## 4. Conclusions

The current study demonstrates that the leaf samples of twelve *Cotoneaster* species cultivated in Poland possess significant and dose-dependent *in vitro* SET- and HAT-type antioxidant activities, which positively correlate with their total phenolic content (TPC). Among the *Cotoneaster* phenolics, the proanthocyanidins were found to be primarily responsible for the tested activity. The leaves of *C. zabelii*, *C. splendens*, *C. bullatus*, *C. divaricatus*, *C. hjelmqvistii* and *C. lucidus* (samples grouped in the cluster CL-1)*,* presenting the highest phenolic content and antioxidant activity, were selected as the most valuable sources of powerful antioxidants with great potential for the use in pharmaceutical, cosmetic and food industries. However, further detailed studies are needed to explore the molecular structure and antioxidant capacity of their individual phenolic constituents as well as to clarify the possible toxicity and other *in vivo* biological properties of the tested *Cotoneaster* extracts.

## Figures and Tables

**Table 1:** UHPLC-PDA-ESI-QTOF-MS data of identified polyphenols in the *Cotoneaster* leaf extracts.

No. ^a^	Compound	*t_R_* (min)	UV (nm)	[M − H]^−^ *m*/*z*	*C. integerrimus*	*C. tomentosus*	*C. melanocarpus*	*C. lucidus*	*C. divaricatus*	*C. horizontalis*	*C. nanshan*	*C. hjelmqvistii*	*C. dielsianus*	*C. splendens*	*C. bullatus*	*C. zabelii*
**1**	3-*O*-caffeoylquinic acid (NCHA) ^b,c^	12.2	294, 325	353.1	+	+	+	+	+	+	+	+	+	+	+	+
**2**	dicaffeoylquinic acid isomer ^d^	17.8	295, 325	515.1	+	+	+	+	+	+	+	+	+	+	+	+
**3**	5-*O*-caffeoylquinic acid (CHA) ^b,c^	19.7	294, 325	353.1	+	+	+	+	+	+	+	+	+	+	+	+
**4**	4-*O*-caffeoylquinic acid (CCHA) ^b,c^	23.6	294, 325	353.1	+	+	+	+	+	+	+	+	+	+	+	+
**5**	procyanidin dimer B-2 ^c^	26.8	280	577.2	+	+	+	+	+	+	+	+	+	+	+	+
**6**	5-*p*-coumaroylquinic acid ^b^	27.9	289, 310	337.1	+	+	+	+	+	+	+	+	+	+	+	+
**7**	(−)-epicatechin ^c^	30.5	280	289.1	+	+	+	+	+	+	+	+		+	+	+
**8**	caffeic acid derivative ^d^	33.3	290, 328	613.1 ^e^		+	+					+				+
**9**	procyanidin trimer C-1 ^c^	37.8	280	865.2	+	+	+	+	+	+	+	+	+	+	+	+
**10**	procyanidin B-type tetramer ^d^	41.9	280	1153.1				+	+	+	+	+	+	+	+	+
**11**	procyanidin B-type trimer ^d^	43.9	280	865.2				+	+			+		+	+	+
**12**	quercetin 3-*O*-β-glucoside-7-*O*-α-rhamnoside ^c^	44.3	265, 350	609.2						+						+
**13**	procyanidin B-type tetramer ^d^	45.6	280	1153.2				+		+		+				+
**14**	quercetin 3-*O*-β-(2′′-*O*-β-xylosyl)galactoside ^c^	46.3	268, 355	595.1						+	+	+	+	+	+	
**15**	epicatechin derivative ^d^	48.9	280	739.2		+	+	+	+	+	+	+	+	+	+	+
**16**	epicatechin derivative ^d^	50.5	280	739.2		+	+	+	+	+	+	+	+	+	+	+
**17**	quercetin rhamnoside-hexoside ^d^	53.7	265, 350	609.1	+	+	+	+	+	+	+	+	+	+	+	+
**18**	hyperoside ^c^	55.0	265, 355	463.1	+	+	+	+	+	+	+	+	+	+	+	+
**19**	quercetin dirhamnoside ^d^	55.7	275, 345	593.1		+				+						+
**20**	rutin ^c^	56.8	260, 355	609.1	+	+	+	+	+	+	+	+	+	+	+	+
**21**	isoquercitrin ^c^	58.4	275, 350	463.1	+	+	+	+	+	+	+	+	+	+	+	+
**22**	procyanidin B-type dimer ^d^	62.2	280	577.1				+	+			+		+	+	+
**23**	procyanidin B-type trimer ^d^	64.7	280	865.2				+	+			+		+		+
**24**	quercetin hexoside derivative ^d^	65.4	256, 355	505.1	+	+	+		+		+					+
**25**	quercetin hexoside derivative ^d^	65.6	256, 355	505.1	+	+	+				+					
**26**	kaempferol rhamnoside-hexoside ^d^	66.5	273, 345	593.1	+	+	+	+	+	+		+		+		+
**27**	quercetin rhamnoside-hexoside ^d^	66.6	276, 350	609.1		+	+	+	+	+			+	+	+	
**28**	quercitrin ^c^	67.3	276, 350	447.1	+	+	+	+	+	+		+	+	+	+	+
**29**	dicaffeoylquinic acid isomer ^d^	67.8	285, 325	515.1	+	+	+	+	+	+	+	+	+	+		
**30**	quercetin hexoside derivative ^d^	69.2	255, 355	505.1	+	+										+
**31**	dicaffeoylquinic acid isomer ^d^	70.9	286, 325	515.1	+	+	+		+	+	+	+	+	+		+
**32**	unknown compound	71.8	280	451.1	+	+	+	+	+	+	+	+	+	+	+	+
**33**	kaempferol rhamnoside-hexoside ^d^	72.5	275, 345	593.1		+						+	+	+		+
**34**	unknown compound	75.8	316	487.3	+	+	+	+	+	+	+	+	+	+	+	+

^a^ peak number and retention time refer to Figure 1; ^b^ identified based on the published literature; ^c^ identified with the corresponding standards; ^d^ tentative assignment based on MS and UV-Vis spectra; ^e^ [M + Na − 2H]^−^.

**Table 2:** Total phenolic (TPC), total proanthocyanidin (TPAC), and total flavonoid (TFC) contents in the *Cotoneaster* leaf extracts ^a^.

No.	Leaf Sample	TPC (% GAE)	TPAC (% CYE)	TFC (%)	
				QU	KA
**1**	*C. integerrimus*	8.74 ± 0.38 ^C^	5.59 ± 0.05 ^A^	1.32 ± 0.04 ^H^	0.073 ± 0.003 ^G^
**2**	*C. tomentosus*	5.17 ± 0.12 ^A^	2.60 ± 0.01 ^D^	0.36 ± 0.01 ^B^	0.097 ± 0.004 ^H^
**3**	*C. melanocarpus*	5.48 ± 0.07 ^A^	2.14 ± 0.03 ^C^	0.26 ± 0.01 ^A^	0.025 ± 0.001 ^B^
**4**	*C. lucidus*	10.68 ± 0.10 ^D^	6.34 ± 0.12 ^F^	0.40 ± 0.01 ^B,C^	0.027 ± 0.001 ^B,C^
**5**	*C. divaricatus*	11.97 ± 0.07 ^E^	9.08 ± 0.04 ^B^	0.70 ± 0.03 ^D^	0.049 ± 0.001 ^E^
**6**	*C. horizontalis*	7.30 ± 0.20 ^B^	5.36 ± 0.04 ^A^	0.27 ± 0.01 ^A^	0.127 ± 0.003 ^D^
**7**	*C. nanshan*	8.43 ± 0.17 ^C^	4.13 ± 0.05 ^E^	0.97 ± 0.02 ^G^	nd
**8**	*C. hjelmqvistii*	12.49 ± 0.41 ^E,F^	9.40 ± 0.36 ^B^	0.43 ± 0.03 ^C^	0.035 ± 0.001 ^C^
**9**	*C. dielsianus*	6.99 ± 0.09 ^B^	5.69 ± 0.05 ^A^	0.52 ± 0.02 ^E^	0.259 ± 0.006 ^I^
**10**	*C. splendens*	9.92 ± 0.33 ^D^	9.13 ± 0.12 ^B^	0.73 ± 0.02 ^D^	0.126 ± 0.005 ^D^
**11**	*C. bullatus*	15.43 ± 0.51 ^G^	14.98 ± 0.08 ^H^	0.61 ± 0.01 ^F^	nd
**12**	*C. zabelii*	12.94 ± 0.28 ^F^	10.86 ± 0.09 ^G^	0.28 ± 0.01 ^A^	0.063 ± 0.001 ^F^

^a^ All values are presented as the means ± standard deviation (SD) calculated per dw of the plant material (*n* = 3 × 5 × 1); different capital letters within the same row indicate significant differences at *p* < 0.05 by Tukey’s test; TPC, total phenolic content, expressed as Gallic acid equivalents (GAE); TPAC, total proanthocyanidin content, expressed as cyanidin chloride equivalents (CYE); TFC, total flavonoid content, quantified by HPLC; QU, quercetin; KA, kaempferol.

**Table 3:** Total content of chlorogenic acid isomers (CHAC) in the *Cotoneaster* leaf extracts ^a^.

No.	Leaf Sample	CHAC (%)		
		NCHA	CHA	CCHA
**1**	*C. integerrimus*	0.125 ± 0.001 ^A^	1.58 ± 0.01 ^B^	0.058 ± 0.001 ^A^
**2**	*C. tomentosus*	0.069 ± 0.003 ^C,D^	0.68 ± 0.03 ^A^	0.037 ± 0.002 ^B^
**3**	*C. melanocarpus*	0.081 ± 0.002 ^D^	0.65 ± 0.02 ^A^	0.031 ± 0.001 ^E^
**4**	*C. lucidus*	0.170 ± 0.008 ^B^	1.19 ± 0.05 ^E^	0.063 ± 0.002 ^C,D^
**5**	*C. divaricatus*	0.327 ± 0.005 ^E^	1.70 ± 0.04 ^C^	0.099 ± 0.004 ^F^
**6**	*C. horizontalis*	0.108 ± 0.003 ^A^	1.57 ± 0.04 ^B^	0.047 ± 0.001 ^A^
**7**	*C. nanshan*	0.116 ± 0.001 ^A^	1.75 ± 0.01 ^C^	0.053 ± 0.001 ^A^
**8**	*C. hjelmqvistii*	0.166 ± 0.005 ^B^	2.70 ± 0.01 ^G^	0.067 ± 0.003 ^C^
**9**	*C. dielsianus*	0.103 ± 0.003 ^A^	0.94 ± 0.02 ^D^	0.039 ± 0.001 ^B^
**10**	*C. splendens*	0.167 ± 0.008 ^B^	1.39 ± 0.06 ^F^	0.040 ± 0.002 ^B^
**11**	*C. bullatus*	0.161 ± 0.003 ^B^	0.63 ± 0.01 ^A^	0.068 ± 0.001 ^C^
**12**	*C. zabelii*	0.066 ± 0.001 ^C^	0.57 ± 0.01 ^A^	0.051 ± 0.001 ^A^

^a^ All values are presented as the means ± SD calculated per dw of the plant material (*n* = 3 × 5 × 1); different capital letters within the same row indicate significant differences at *p* < 0.05 by Tukey’s test; CHAC, content of chlorogenic acid isomers quantified by HPLC; NCHA, neochlorogenic acid; CHA, chlorogenic acid and CCHA, cryptochlorogenic acid.

**Table 4:** Correlation (*r*) and determination (*R*^2^) coefficients for linear relationships between antioxidant capacities and phenolic contents of the *Cotoneaster* leaf extracts ^a,b^.

*r* (*R*^2^)	DPPH EC_50_ (µg/mL)	FRAP (mmolFe^2+^/g)	O_2_^•−^ SC_50_ (µg/mL)	H_2_O_2_ SC_50_ (µg/mL)
**TPC (% GAE)**	−0.8298 (0.6885) *	0.9491 (0.9008) *	−0.4227 (0.1787)	−0.8676 (0.7528) *
**TPAC (% CYE)**	−0.7706 (0.5938) **	0.9719 (0.9447) *	−0.2854 (0.0815)	−0.7639(0.5836) **
**TFC (%; QU + KA)**	−0.1907 (0.0364)	0.0355 (0.0013)	−0.0976 (0.0095)	−0.0529 (0.0028)
**CHAC (%; NCHA + CHA + CCHA)**	−0.5366 (0.2879)	0.1392 (0.0194)	−0.4062 (0.1650)	−0.3148 (0.0991)
**DPPH EC_50_ (µg/mL)**	–	−0.8364 (0.6996) *	0.4218 (0.1779)	0.7580 (0.5746)**
**FRAP (mmol Fe^2+^/g)**	−0.8364 (0.6996) *	–	−0.3490 (0.1218)	−0.7746 (0.6000) **
**O_2_^•−^ SC_50_ (µg/mL)**	0.4218 (0.1779)	−0.3490 (0.1218)	–	0.7127 (0.5079) **
**H_2_O_2_ SC_50_ (µg/mL)**	0.7580 (0.5746) **	−0.7746 (0.6000) **	0.7127 (0.5079) **	–

^a^ For activity and quantitative parameters, see Table 2 and Table 3, and Figure 3; ^b^ significance levels of * *p* < 0.001, ** *p* < 0.01.

**Table 5:** Herbarium codes and collection sites of the analyzed *Cotoneaster* Medik. species.

No.	Species	Herbarium Code	Collection Site
**1**	*C. integerrimus* Medik.	KFG/12/CIN	Forestry Experimental Station of Warsaw University of Life Sciences (Rogow, Poland)
**2**	*C. tomentosus* Lindl.	KFG/12/CTM	
**3**	*C. melanocarpus* Lodd. ex. C.K. Schneid.	KFG/12/CMA	
**4**	*C. lucidus* Schltdl.	KFG/12/CLC	
**5**	*C. divaricatus* Rehder et E.H. Wilson	KFG/12/CDV	Botanical Garden (Lodz, Poland)
**6**	*C. horizontalis* Decne.	KFG/12/CHR	
**7**	*C. nanshan* Mottet	KFG/12/CNA	
**8**	*C. hjelmqvistii* Flinck et B. Hylmö	KFG/12/CHQ	
**9**	*C. dielsianus* E. Pritz.	KFG/12/CDL	
**10**	*C. splendens* Flinck et B. Hylmö	KFG/12/CSP	
**11**	*C. bullatus* Bois	KFG/12/CBL	
**12**	*C. zabelii* C.K. Schneid	KFG/12/CZB	

## Supplementary Materials

Supplementary materials can be accessed at: http://www.mdpi.com/1420-3049/21/6/688/s1.
