# Supplementary material for: Phenolic Profile and Antioxidant Potential of Leaves from Selected Cotoneaster Medik. Species
Source: Molecules. 2016 May 26;21(6):688. doi: 10.3390/molecules21060688 (PMC6274135; doi:10.3390/molecules21060688)
Supplement: Supplementary file 1 [file molecules-21-00688-s001.pdf]

Supplementary Materials: Phenolic Profile and Antioxidant Potential of Leaves from Selected *Cotoneaster* Medik. Species

Agnieszka Kicel, Piotr Michel, Aleksandra Owczarek, Anna Marchelak, Dorota Żyżelewicz, Grażyna Budryn, Joanna Oracz and Monika Anna Olszewska

Table S1. Retention times, UV-Vis and QTOF-MS data in the *Cotoneaster* leaf extracts.

No[a]	Compound	t_R (min)	UV (nm)	Formula	$[M-H]^-$ *m/z*	MS Fragments (% relative abundance)
1	3-*O*-caffeoylquinic acid (NCHA) [b,c]	12.2	294, 325	$C_{16}H_{18}O_9$	353.1	191.0 (100), 179.0 (55)
2	dicaffeoylquinic acid isomer [d]	17.8	295, 325	$C_{25}H_{24}O_{12}$	515.1	353.1 (6)
3	5-*O*-caffeoylquinic acid (CHA) [b,c]	19.7	294, 325	$C_{16}H_{18}O_9$	353.1	191.0 (100), 179.0 (4)
4	4-*O*-caffeoylquinic acid (CCHA) [b,c]	23.6	294, 325	$C_{16}H_{18}O_9$	353.1	191.0 (40), 179.0 (50), 173.0 (100)
5	procyanidin dimer B-2 [c]	26.8	280	$C_{30}H_{26}O_{12}$	577.1	451.1 (5), 425.1 (20), 407.0 (22), 289.0 (8)
6	5-*p*-coumaroylquinic acid [b]	27.9	289, 310	$C_{16}H_{18}O_8$	337.1	191.0 (88), 163.0 (5)
7	(−)-epicatechin [c]	30.5	280	$C_{15}H_{14}O_6$	289.0	245.0 (6), 205.0 (8)
8	caffeic acid derivative [d]	33.3	290, 328		613.1[e]	591.1 (45), 295.6 (100), 179.0 (5)
9	procyanidin trimer C-1 [c]	37.8	280	$C_{45}H_{38}O_{18}$	865.2	713.1 (16), 577.1 (17), 451.1 (7), 407.0 (12), 289.0 (4)
10	procyanidin B-type tetramer [d]	41.9	280	$C_{60}H_{50}O_{24}$	1153.1	1027.2 (20), 863.2 (69), 757.2 (17), 575.1 (95), 407.0 (19), 289.0 (12)
11	procyanidin B-type trimer [d]	43.9	280	$C_{45}H_{38}O_{18}$	865.2	713.1 (32), 575.1 (38), 451.1 (9), 407.0 (13), 289.0 (6)
12	quercetinn 3-*O*-β-glucoside-7-*O*-α-rhamnoside [c]	44.3	265, 350	$C_{27}H_{30}O_{16}$	609.1	446.1 (50), 299.0 (6)
13	procyanidin B-type tetramer [d]	45.6	280	$C_{60}H_{50}O_{24}$	1153.1	1027.1 (18), 863.2 (60), 575.1 (86), 449.1 (61), 407.0 (100), 289.0 (81)
14	quercetin 3-*O*-β-(2″-*O*-β-xylosyl)galactoside [c]	46.3	268, 355	$C_{26}H_{28}O_{16}$	595.1	300.0 (42)
15	epicatechin derivative [d]	48.9	280		739.2	587.1 (26), 449.1 (12), 435.0 (6), 339.0 (12), 289.0 (11)
16	epicatechin derivative [d]	50.5	280		739.2	587.1 (19), 575.1 (8), 449.1 (15), 407.1 (6), 339.0 (9), 289.0 (13)
17	quercetin rhamnoside-hexoside [d]	53.7	265, 350	$C_{27}H_{30}O_{16}$	609.1	300.0 (6)
18	quercetin 3-*O*-β-galactoside (hyperoside) [c]	55.0	265, 355	$C_{21}H_{20}O_{12}$	463.1	300.0 (19)
19	quercetin dirhamnoside [d]	55.7	275, 345	$C_{27}H_{30}O_{15}$	593.1	446.1 (74), 299.0 (10)
20	3-*O*-β-(6″-*O*-α-rhamnosyl)glucoside (rutin) [c]	56.8	260, 355	$C_{27}H_{30}O_{16}$	609.1	300.0 (26)

Table S1. *Cont.*

No[a]	Compound	t_R (min)	UV (nm)	Formula	$[M-H]^-$ *m/z*	MS Fragments (% relative abundance)
21	quercetin 3-*O*-β-glucoside (isoquercitrin) [c]	58.4	275, 350	$C_{21}H_{20}O_{12}$	463.1	300.0 (31)
22	procyanidin B-type dimer [d]	62.2	280	$C_{30}H_{26}O_{12}$	577.1	451.2 (9), 425.1 (23), 407.0 (22), 289.0 (17)
23	procyanidin B-type trimer [d]	64.7	280	$C_{45}H_{38}O_{18}$	865.2	739.1 (21), 713.1 (22), 577.1 (43), 451.1 (19), 407.0 (23), 289.0 (12)
24	quercetin hexoside derivative [d]	65.4	256, 355		505.1	463.1 (3), 300.0 (57)
25	quercetin hexoside derivative [d]	65.6	256, 355		505.1	463.1 (2), 300.0 (35)
26	kaempferol rhamnoside-hexoside [d]	66.5	273, 345	$C_{27}H_{30}O_{15}$	593.1	285.0 (15)
27	quercetin rhamnoside-hexoside [d]	66.6	276, 350	$C_{27}H_{30}O_{16}$	609.1	300.0 (32)
28	quercetin 3-*O*-β-rhamnoside (quercitrin) [c]	67.3	276, 350	$C_{21}H_{20}O_{11}$	447.1	300.0 (21)
29	dicaffeoylquinic acid isomer [d]	67.8	285, 325	$C_{25}H_{24}O_{12}$	515.1	353.1 (100), 335.0 (3)
30	quercetin hexoside derivative [d]	69.2	245, 345		505.1	463.1 (4), 300.0 (58)
31	dicaffeoylquinic acid isomer [d]	70.9	286, 325	$C_{25}H_{24}O_{12}$	515.1	353.1 (16), 255.0 (2)
32	unknown compound	71.8	280		451.1	373.1 (4), 341.0 (24)
33	kaempferol rhamnoside-hexoside [d]	72.5	275, 345	$C_{27}H_{30}O_{15}$	593.1	447.0 (20), 285.0 (11)
34	unknown compound	75.8	316		487.3	469.3 (8), 443.3 (4)

[a] peak number and retention time refer to Figure 1; [b] identified based on the published literature; [c] identified with the corresponding standards; [d] tentative assignment based on MS and UV-Vis spectra; [e] $[M + Na - 2H]^-$
